# Role of Dental Professionals in the Management of a Dentigerous Cyst in a Patient With Amelogenesis Imperfecta

**DOI:** 10.7759/cureus.32696

**Published:** 2022-12-19

**Authors:** Suresh Chinnakutti, Karthik Rajaram Mohan, Devaki Murugesan, Sarathchandra Govind Raj, Akash Mithran

**Affiliations:** 1 Oral and Maxillofacial Surgery, Vinayaka Missions Sankarachariyar Dental College, Vinayaka Missions Research Foundation (Deemed to be University), Salem, IND; 2 Oral Medicine and Radiology, Vinayaka Missions Sankarachariyar Dental College, Vinayaka Missions Research Foundation (Deemed to be University), Salem, IND; 3 Pedodontics and Preventive Dentistry, Kanyakumari Government Medical College, Nagercoil, IND; 4 Prosthodontics, Rajas Dental College and Hospital, Kavalkinaru, IND

**Keywords:** indian dentist, amelogenesis imperfecta, marsupilization, dentists, impacted tooth, dentigerous cyst

## Abstract

Amelogenesis imperfecta (AI) is a hereditary condition that affects the clinical features and structure of the enamel. The enamel formation diseases are inherited and might be X-linked, autosomal dominant, recessive, sex-related, or sporadic. Dental professionals should evaluate such patients completely, both clinically and radiographically, to detect any cysts associated with impacted or unerupted teeth. This report describes the case of a 10-year-old patient affected by amelogenesis imperfecta with a dentigerous cyst involving an impacted tooth and an unerupted tooth of the maxilla.

## Introduction

The term "amelogenesis imperfecta" (AI) refers to a group of hereditary illnesses that primarily affect the enamel of all or nearly all of the teeth without manifesting in any other parts of the body [1]. X-linked, autosomal dominant, recessive, sex-related, or sporadic are possible causes [2]. According to the findings, AI occurs between 1:718 and 1:14,000 times per year [3, 4]. It is always associated with multiple impacted and unerupted teeth; dentigerous cysts are common with impacted teeth. There are currently at least 15 subtypes of AI based on the phenotypic form and inheritance pattern. There are four recognised phenotype subtypes of AI, including taurodontism and combined hypomature/hypoplastic and hypocalcified phenotypes. It has always been linked to numerous impacted and unerupted teeth. Both primary and permanent teeth are involved in AI. AI related to delayed eruption and impacted teeth has been discussed in a small number of case reports in the literature [5, 6]. About 16% to 24% of all genuine cysts involving the crown of an impacted tooth are dentigerous cysts, the second most frequent odontogenic cysts [7,8]. Individuals with AI frequently visit dental offices for cosmetic procedures. If we focus solely on aesthetic issues, we risk missing pathologies such as cysts related to impacted or unerupted teeth at an early stage.

## Case presentation

**Figure 1:**
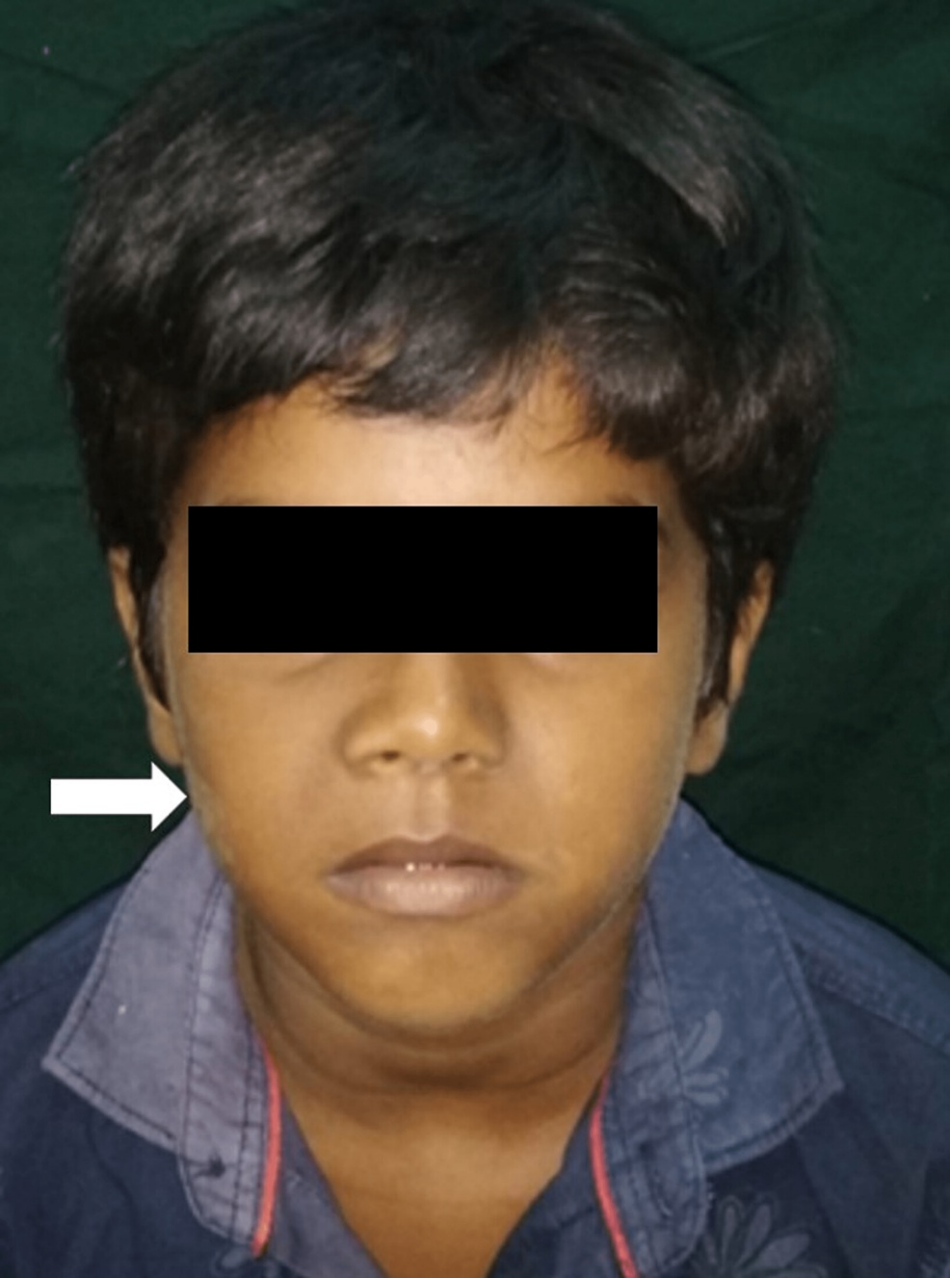
Extraoral examination revealed swelling on the right middle third of the face.

A 10-year-old boy came with his parents with a chief complaint of swelling in the right cheek region for the past two months. The swelling was small in size initially, gradually increased, and was not associated with pain. He also complains of the accidental chipping of a tooth during the mastication of foods. His parents had a consanguineous marriage. His elder brother had normal dentition. There is no other relevant medical or family history. Extraoral examination revealed a 2 x 2 cm diffused swelling with tenderness in the right middle third of the face (Figure 1).

**Figure 2:**
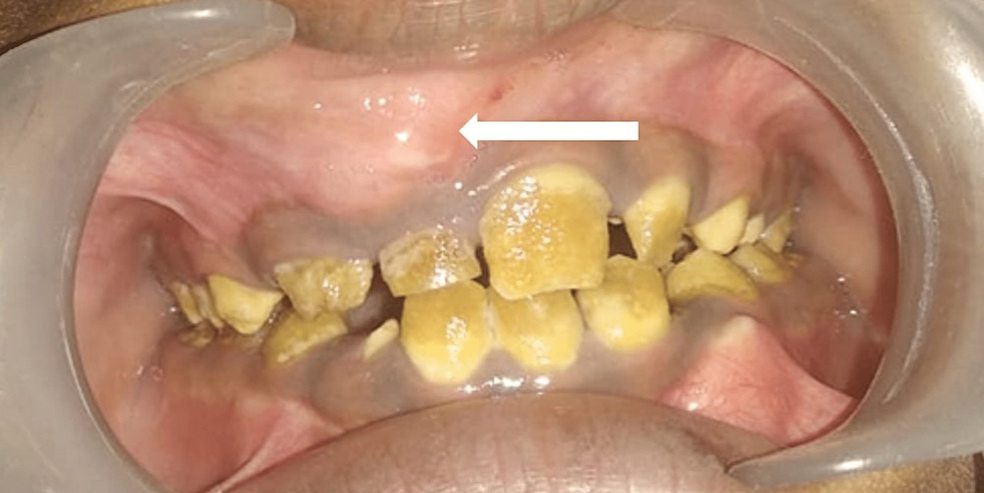
Intraoral examination revealed obliteration of the labial vestibule in relation to the 51-tooth region and generalised yellowish intrinsic discolouration of all the teeth.

Intraoral examination revealed obliteration of the labial vestibule in relation to 51, 12, and 14 regions with firmness in consistency, tenderness, and labial cortex expansion (Figure 2). Intraoral hard tissue examination revealed yellowish discolouration in the crown of all the teeth and the presence of intact enamel margins only near the cementoenamel junction near the marginal gingiva of all the teeth.

**Figure 3:**
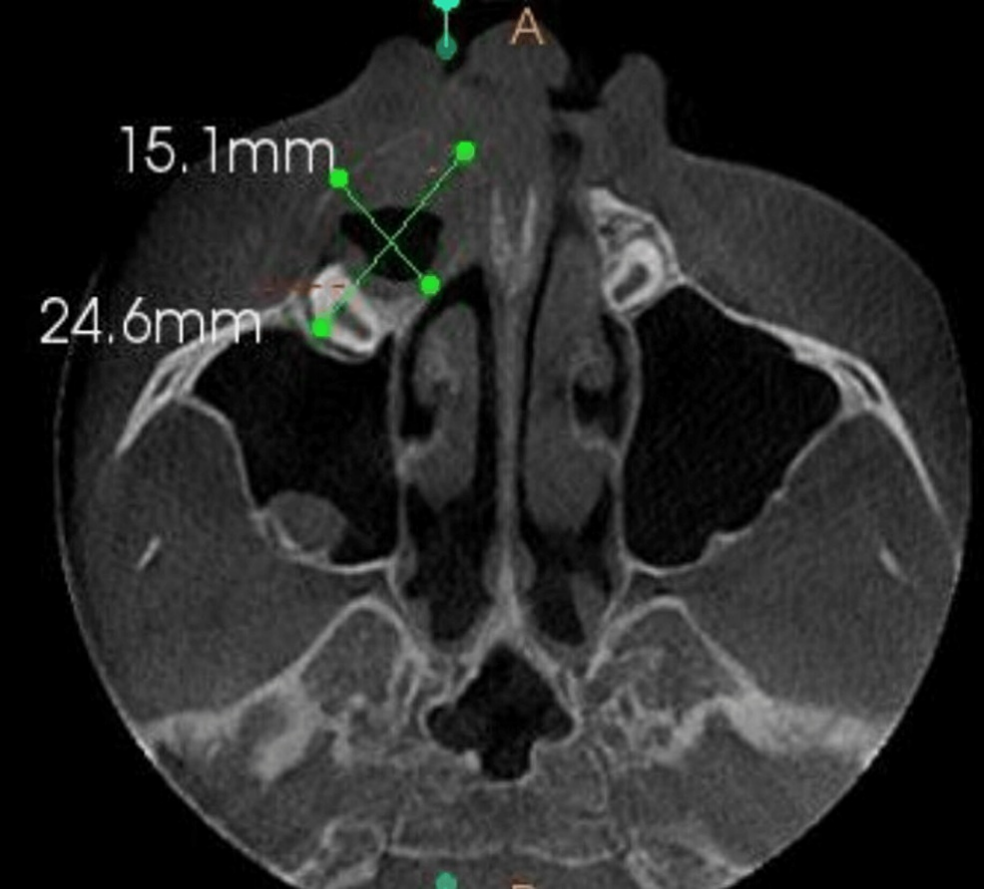
CBCT (axial section) revealed an expansile hypodense area measuring 15.1 mm by 24.6 mm around the crown of an impacted right maxillary canine tooth 13 in relation to the anterior-superior wall of the floor of the right maxillary sinus.

Primary and permanent teeth were both brownish yellow in colour, with intact enamel near the cervical third of the crowns of retained deciduous teeth 51 and permanent teeth 12, 13, 21, 22, 23, 31, 32, and 41. Based on these clinical findings and the history of chipped teeth, a provisional diagnosis of amelogenesis imperfecta was made. The patient's facial profile was straight. A pre-operative cone-beam computed tomography scan (CBCT) was taken, in which an axial section revealed a hypodense area measuring 15.1 mm by 24.6 mm around the impacted right maxillary canine 13 in relation to the anterior-superior wall of the floor of the right maxillary sinus ( Figure 3).

**Figure 4:**
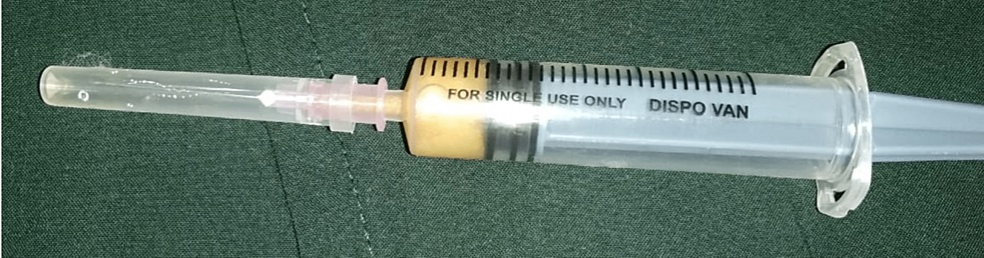
Aspiration revealed yellowish-coloured fluid.

Following haematological examinations, a yellowish viscous fluid was aspirated from the swelling (Figure 4).

**Figure 5:**
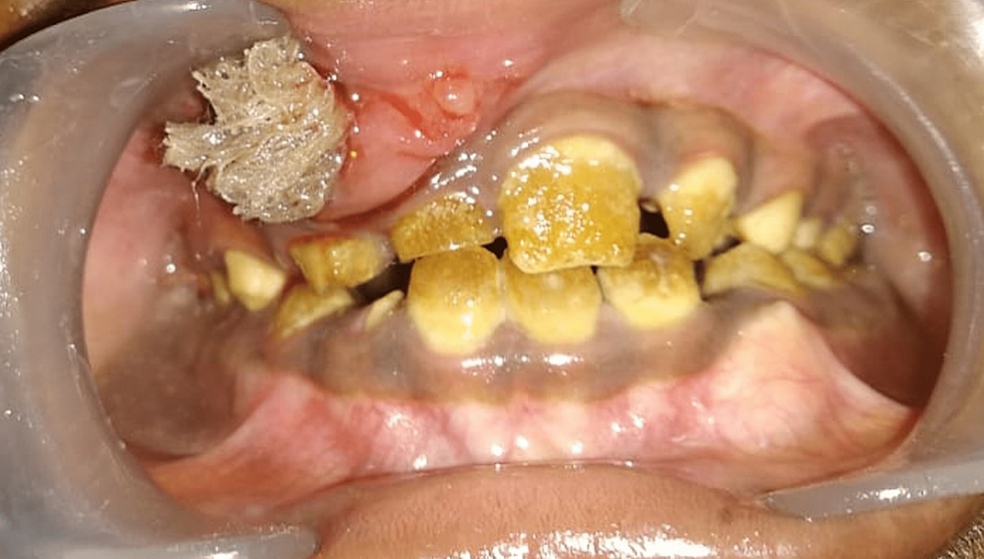
Gauze impregnated with Bismuth Iodoform Paraffin Paste (BIPP) after the marsupilization procedure.

Treatment was planned as an intraoral approach, with marsupialization to preserve unerupted tooth 13, an impacted 11 tooth, and Bismuth Iodoform Paraffin Paste (BIPP) pack placement under local anaesthesia. Specimens were sent for histopathological examination. The biopsy report revealed a cystic lesion with a non-keratinised epithelial lining of 2-4 cell layers, a connective tissue wall with chronic inflammatory infiltrate and haemorrhage, and concluded with a dentigerous cyst. The patient is under regular follow-up with BIPP pack replacement at regular intervals for six weeks ( Figure 5).

**Figure 6:**
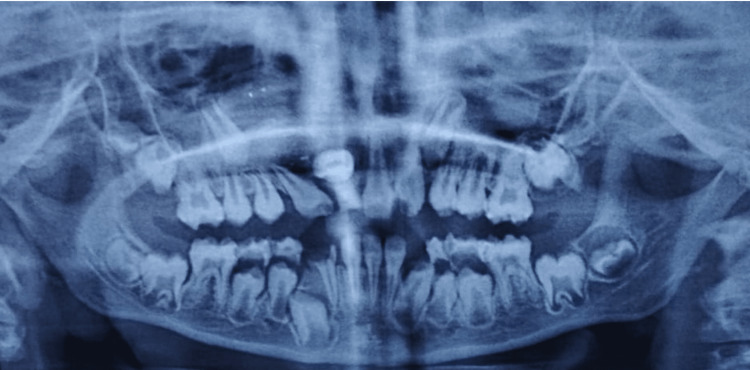
A panoramic radiograph after six weeks, during the postoperative follow-up, revealed the complete shrinkage of the lesion.

A post-operative panoramic radiograph was taken after six weeks, which revealed the lesion's evident shrinkage and radiodensity of enamel, the same as that of the underlying dentin on teeth 14, 15, 16, 17, 22, 23, 24, 25, 26, 31, 32, 46, and 47 ( Figure 6).

**Figure 7:**
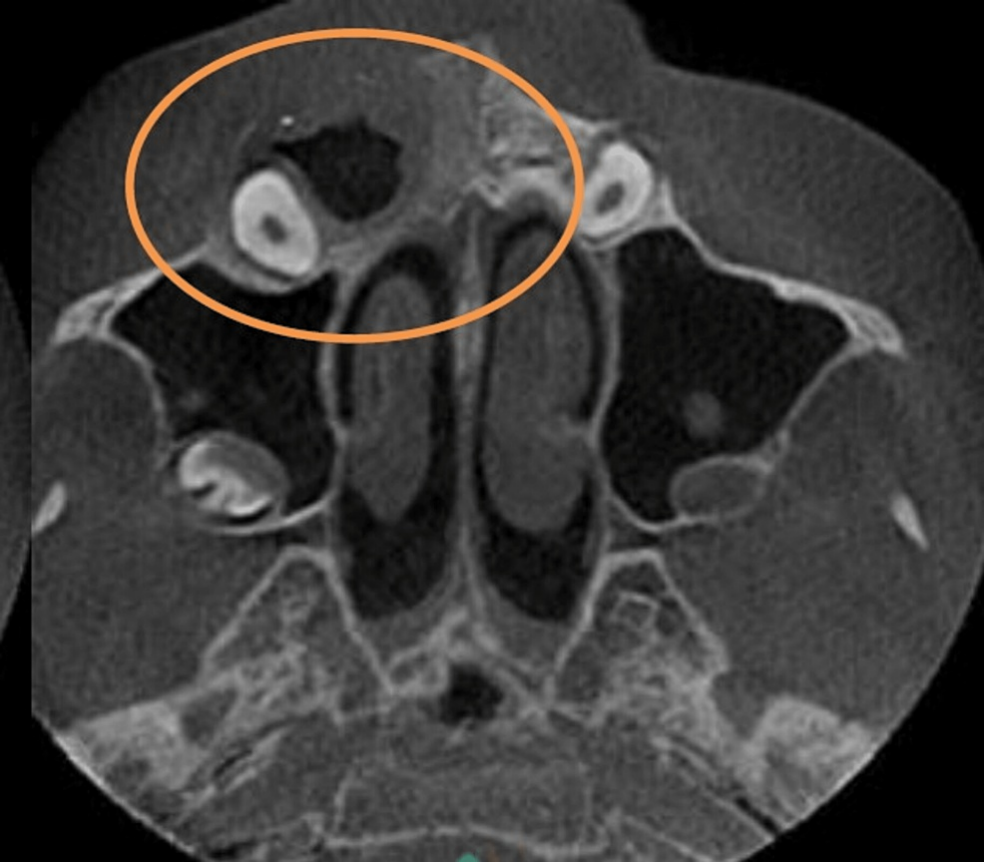
CBCT (axial section) revealed shrinkage of the hypodense area within the impacted right maxillary canine 13.

A follow-up with CBCT also showed shrinkage of the lesion around the impacted right maxillary canine 13 [Figure 7]. 

## Discussion

 

Qualitative and quantitative enamel deficiencies are among the dental characteristics linked to AI, along with other characteristics such as root and crown resorption, taurodontism, pulpal calcification, malformation of the root, impaction of permanent teeth, and congenitally absent teeth [6]. In our situation, we have an impacted upper right central incisor with a cystic lesion, loss of enamel on the crown, etc. In addition to being concerned about facial swelling owing to the underlying cystic lesion, our patient was also concerned about AI's marked effects on children and adolescents in the aspects of aesthetics, function, and psychological disturbance due to discoloured teeth, as noted by Parekh S et al [9]. Patil et al. reported a case of AI with multiple impacted teeth, and Hegde et al. reported a case with multiple unerupted teeth, but our case had both unerupted teeth and impacted teeth with cysts [4, 10]. Dentigerous cysts associated with impacted teeth in AI patients are rarely reported in the literature. During the mixed dentition period, Möhn M. et al. discussed various management dentitions for AI patients [11]. Matthew L. et al. and Tripathy M. et al. reported a case of multiple periapical cysts in an AI patient and its management. Our patient was also in a mixed dentition period with a dentigerous cyst [12, 13]. Our primary concern was also to manage cystic lesions. The mentioned evidence from the literature emphasises that AI patients with impacted or unerupted teeth are more susceptible to cystic changes. In our case, the patient's brother had normal dentition. Since AI is inherited as an autosomal recessive pattern, he carried one copy of the affected gene and did not show signs of the condition.

Marsupialization or Partsch I surgery entails making a split in a cyst's wall and stitching the slit's borders together to create a continuous surface that extends from the cyst's exterior to its interior surface. The site will remain open and be able to drain freely after being sutured in this way. The use of BIPP-impregnated gauze in the cystic cavity ensures little nutrition for bacteria to thrive in its interstices by forming an impervious barrier to blood and body fluids.

In 1916, James Rutherford Morrison, Professor of Surgery at Durham University and Surgeon-in-Charge, at Northumberland General Hospital, Newcastle invented the BIPP as a medicament to treat infection in the cavities of wounded soldiers during World War I [14]. It is made of liquid paraffin, two parts iodoform, and one part bismuth subnitrate or carbonate in an iodoform base. BIPP is made up of the following chemical components: iodoform (40% by weight), bismuth subnitrate (20%), and paraffin liquid (40% by weight). It has numerous uses, particularly in the fields of oral and maxillofacial surgery, ear, nose, and throat surgery, and neurosurgery. Using BIPP, impregnated gauze becomes resistant to blood and bodily fluids, providing little nutrition for bacteria to grow in its crevices. Bismuth can be used as an astringent and has topical antiseptic effects. Iodoform (also known as triiodomethane) hydrolysis produces diluted nitric acid, which contributes to BIPP's antibacterial properties. This is an additional element of BIPP. Both its hue and scent are distinctive. Iodine, an antiseptic, is released as iodoform breaks down. As a lubricant, paraffin is added to the BIPP to help with the atraumatic placement and removal of the pack. It can also activate the granulation tissue. In our experience, its resilience amid necrotic tissue, producing clean, controllable cavities, is outstanding. Since the defect was too large to hold the sutures placed during initial closure, BIPP was employed as a dressing.

The cystic cavity was filled with ribbon gauze covered in bismuth iodide paste. The dressing was changed once every seven to 10 days for the first three months until radiographically adequate healing was seen [15]. During computed tomography scans, the high attenuation coefficient of BIPP, greater than 3000 Hounsfield Units (HU), might cause streak artefacts. Therefore, it is recommended that BIPP dressings be removed before the patient is subjected to computed tomography scans [16]. O'Connor AF reported iodoform toxicity when BIPP was used in packing large defects caused by maxillectomy [ 17]. Wilson stated that BIPP, which is used to fill cavities in the mandible, can cause personality changes and insomnia due to encephalopathy after more than seven weeks of use [18]. The sensitivity to bismuth sulphate or iodoform in bismuth iodoform paraffin paste (BIPP) is type IV delayed hypersensitivity reaction, largely involving T cells and macrophages. BIPP also aids in controlling infection and healing following surgical debridement or sequestrectomy of bone affected by osteomyelitis [19]. Instead of laminate veneers, zirconia-based full ceramic or all-ceramic crowns are recommended for prosthodontic rehabilitation of teeth in patients with amelogenesis imperfecta, because veneer marginal adaptation is difficult due to enamel chipping [20].

## Conclusions

Dental professionals must be careful while treating any patients with clinical features of amelogenesis imperfecta (AI). A thorough dental examination is required, and if any teeth are missing, we should recommend a radiograph for early diagnosis of any cystic lesion associated with impacted or unerupted teeth. These patients should also be followed up regularly to avoid morbidity and reduce the psychosocial impact on the patient and parents. Zirconia-based restorations or metal-ceramic crowns are more effective than porcelain laminate veneers at restoring masticatory function and improving aesthetics when marginal adaptation fails because of enamel chipping close to the delicate cervical cemento-enamel junction of teeth.
